# Home environment: Short‐term trends and predictors in early childhood from an Indian community birth cohort

**DOI:** 10.1111/cch.12846

**Published:** 2021-01-20

**Authors:** Beena Koshy, Arun Karthikeyan, Anuradha Bose, Reeba Roshan, Karthikeyan Ramanujam, Venkata Raghava Mohan, Sushil John, Gagandeep Kang

**Affiliations:** ^1^ Developmental Paediatrics Unit Christian Medical College Vellore India; ^2^ Wellcome research Unit Christian Medical College Vellore India; ^3^ Community Health Christian Medical College Vellore India; ^4^ Low Cost Effective Care Unit Christian Medical College Vellore India

**Keywords:** early childhood, home environment, maternal education, socio‐economic status

## Abstract

**Background:**

Early childhood home environment is intricately linked to child development and later cognitive and academic skills. There is limited literature evaluating home environmental trends and predictors in the low‐ and middle‐income country settings.

**Aims:**

Determine the trends of early childhood home environment changes between 6 and 36 months of age, and the factors associated with these changes.

**Study design:**

Longitudinal community‐based birth cohort follow‐up study in a semi‐urban slum in Vellore, South India.

**Subjects:**

Consecutive sampling of a birth cohort between March 2010 and February 2012.

**Outcome measures:**

Home environment was objectively assessed using the ‘Home Observation for the Measurement of the Environment’ (HOME) scale. Predictors of change in the home environment over time also were analyzed. Multivariable linear regression models and linear mixed effect models were used to identify the factors associated with HOME score at individual time points and over‐a‐time period, respectively.

**Results:**

The birth cohort enrolled 251 children with a follow‐up of 235, 228 and 218 children at 6, 24 and 36 months, respectively. The socio‐economic status (SES) was the single biggest predictor for the HOME score at each time point, with increasing strength over time. Maternal education predicted home environment at 24 months, while maternal depression was negatively associated at 6 and 24 months of age. SES and maternal factors contributed to the overall change in the HOME score. Maternal factors predicted relational home environmental change over time.

**Conclusion:**

SES and maternal factors consistently predicted early childhood home environment at 6, 24 and 36 months of age and its change over time. It is important to support maternal education and wellbeing along with socio‐economic measures to optimize early childhood environment.

**Key message**

- Maternal and socio‐economic factors were associated with early childhood home environment and its change in this Indian birth cohort follow‐up study
- Maternal factors predicted relational home environmental change over time.
- Maternal well‐being needs to be enquired and supported at well‐baby clinics
- Maternal education should be supported for holistic socio‐economic development planning.

## 1 INTRODUCTION

Early childhood development is intricately linked to the quality of home environment, where the infant and later toddler spends his/her time. Optimal early childhood environments can augment child development, and consecutively child cognitive ability, impacting long‐term individual potential (Andrade et al., 2005; Black et al., 2013; Bradley & Corwyn, 2005; Bradley, Corwyn, & Whiteside‐Mansell, 1996; Molfese, Modglin, & Molfese, 2003; Van Doorninck, Caldwell, Wright, & Frankenburg, 1981). Evaluating child's environment for developmental optimisation is in concurrence with the ‘ecological systems theory’ proposed by Bronfenbrenner, who stressed on the importance of evaluating individual development and life progress in the context of environment (Bronfenbrenner, 1977).

Recent developments stressing the importance of the first thousand days of life on human potential have led to an increased interest, to understand early childhood environment (Biesalski, 2016). A stimulating and quality home environment can independently impact, mediate and modulate child development (Andrade et al., 2005; Prado & Dewey, 2014). Parental cognitive stimulation is associated with early childhood ability and is transactional with genetic influences (Tucker‐Drob & Harden, 2012). Appropriate psycho‐social stimulation improves both growth as well as cognitive ability, as evidenced by studies in Indonesia (Helmizar, Jalal, Lipoeto, & Achadi, 2017), Vietnam (Watanabe, Flores, Fujiwara, & Tran, 2005), Malaysia (Nurliyana, Shariff, Taib, Gan, & Tan, 2020), and Jamaica (Walker, Grantham‐McGregor, Powell, & Chang, 2000). In addition, early life experiences in a quality home environment can not only optimize later childhood academic potential but also augment individual resilience for later life (Bradley et al., 1994; Molfese et al., 2003; Van Doorninck et al., 1981). Concurrently, suboptimal home environment can adversely impact child development and behaviours (Grandjean & Landrigan, 2006; Shonkoff, Garner, Siegel, et al., 2012). Home learning environments have been serially evaluated and are found to be associated with the baseline home learning environment status (Toth et al., 2020) and can affect language and cognitive development in children (Rodriguez & Tamis‐LeMonda, 2011).

Home environmental characteristics can be broadly categorized into physical and relational environments (Jones et al., 2017). Physical environment amounts to the physical safety of the environment including building materials used and state of overcrowding. Relational environment is the sensitive and responsive relationship that the child develops with parents and caregivers (Rasheed & Yousafzai, 2015). Physical and relational environments often modulate each other; maternal responsiveness is shown to mediate the effect of overcrowding on childhood cognition (Evans et al., 2010).

Home environmental status and socio‐economic status (SES) of the family are shown to impact and complement each other (Coscia et al., 2001; Crane, 1996; Ronfani, Brumatti, Mariuz, et al., 2015). Families with higher SES are able to provide better and stimulating home environments including a variety of childhood experiences (Yunus & Dahlan, 2013). Meanwhile, home environment is shown to mediate the effect of or exert more influence than SES on child development (Coscia et al., 2001; Ferreira, Godinez, Gabbard, Vieira, & Caçola, 2018; Lohndorf, Vermeer, Carcamo, & Mesman, 2018; Ronfani et al., 2015), making it a proxy marker for early childhood care and education (Iltus, 2006). In addition, maternal factors including cognition, education and depression can impact child cognition, not only through genetic factors but also by optimizing the relational environment (Andrade et al., 2005; Bradley, Corwyn, Burchinal, McAdoo, & García Coll, 2001; Bradley, Corwyn, McAdoo, & García Coll, 2001; Flynn, Chung, Ozer, & Fernald, 2017; Pachter, Auinger, Palmer, & Weitzman, 2006; Ronfani et al., 2015). Maternal self‐esteem also affects the home environment, as shown in a study from Andhra Pradesh, India (Fernandez, Vazir, Bentley, Johnson, & Engle, 2008).

Home environmental studies in India have shown that home environment influences learning needs (Mohite, 1987) and externalizing behaviour (Gill & Kang, 1995). Home inventory also has been translated into Hindi (Kohli, Mohanty, & Kaur, 2005). However, there has been a paucity of data on trends and predictors of early childhood home environment in Indian settings. The current paper aims to address this by analyzing home factors at 6, 24 and 36 months of age, their predictors and factors contributing to their change over time in a semi‐urban slum in South India. Our hypothesis is that SES along with maternal factors will be found associated with home factors at each time point.

## 2 MATERIALS AND METHODS

### 2.1 Study population and methodology

The current study was done as an independent subanalysis of a large multinational prospective, longitudinal birth cohort study ‘The Etiology, Risk Factors and Interactions of Enteric Infections and Malnutrition and the Consequences for Child Health and Development (MAL‐ED) Network’, conducted across eight different countries across the world (Network Investigators, 2014). The MAL‐ED study aimed to evaluate effects of early childhood factors of enteric infections and nutrition on childhood growth and development including cognition. The study site in India was a densely populated semi‐urban slum comprising of Old Town, Salavanpet and neighbouring areas in Vellore, South India (John et al., 2014). For enrolment, the study population of 12 000 was covered. Pregnant women were identified by a door‐to‐door survey and invited to participate in the study. Children were recruited at birth after an informed parental consent by consecutive sampling. The exclusion criteria were existing plans of the family to migrate out of the site during study period, multiple pregnancies, another child already enrolled in the study, medical co‐morbidities in index child and unavailability of mothers to provide necessary informed consent. The information on parity was collected from both history and from available health records of mothers during the recruitment. Children recruited were visited twice a week till their second birthday for active morbidity surveillance as per the MAL‐ED protocol. Subsequent follow‐ups were conducted starting at 3 years of age. The initial birth cohort recruitment was conducted between March 2010 and February 2012 and recruited 251 newborns. The original birth cohort recruitment and subsequent follow‐ups were approved by the Institutional Review Board and Ethics committee of Christian Medical College Vellore, and children were recruited at each stage after informed consent.

## 3 STUDY MEASURES

### 3.1 The HOME scale

The Home Observation for the Measurement of the Environment (HOME) scale (Infant/Toddler version) measures the support and stimulation, the child receives in the home environment and is widely used with good psychometric properties (Caldwell & Bradley, 1984; Elardo & Bradley, 1981). This observed measure specifically evaluates environmental factors that can potentially influence child development such as mother–child interactions including parental responsiveness and opportunities to learn.

The modified version originally adapted for Bangladesh has six subscales consisting of total 48 items: subscales being responsiveness to parent, avoidance of restriction and punishment, organization of the environment, appropriate play materials, parental involvement and variety in daily stimulation (Black et al., 2004). This version was translated, back‐translated and piloted prior to administration in the study. Factor analysis of this modified scale in the main MAL‐ED study conducted in eight international sites revealed a three‐factor structure of emotional and verbal responsivity, clean and safe environment and child cleanliness (Jones et al., 2017).

A trained social worker completed the HOME scale at 6, 24 and 36 months of child's age after observing the home environment for a 45‐ to 60‐min observation period along with supplementary information from a caregiver interview as per the MAL‐ED protocol (Jones et al., 2017; Murray‐Kolb et al., 2014). Visits were made in the morning and responder items were answered by the mother.

### 3.2 The WAMI measure for SES

The WAMI measure is a simplified SES measure that includes access to improved **W**ater and sanitation, **A**ssets, **M**aternal education and total household reported **I**ncome and has been developed during the MAL‐ED study using a variable selection approach with random forests (Psaki, Seidman, Miller, et al., 2014). Water and sanitation included the type of water source and latrine facility, while physical assets such as television, telephone and vehicle were counted. The questionnaire for WAMI was translated to Tamil and back‐translated before administration by a trained field worker. The final measure was calculated from the variables and then converted to a standardized score ranging from 0 to 1. Serial measure of SES using WAMI score was done at 6, 12, 18, 24 and 36 months of child's age, by home visits.

### 3.3 The self‐reporting questionnaire‐20

The Self‐Reporting Questionnaire‐20 (SRQ‐20) was used to assess maternal psychological disturbances at 1, 6, 15, 24 and 36 months of child age (Beusenberg et al., 1994). This measure especially developed by the World Health Organization (WHO) for use in low‐resource settings can assess depressive symptoms. After translation, back‐translation and piloting, the measure was administered at home by a trained social worker.

### 3.4 Raven's progressive matrices

Maternal cognition was assessed at 6–8 months of child age by Raven's progressive matrices (Raven, Raven, & Court, 2003). The Raven's test is a non‐verbal measure of reasoning ability and can be used across cultures. A trained psychologist administered this test in a centrally located distraction‐free room to mothers as per the MAL‐ED protocol and took 20–40 min for administration (Murray‐Kolb et al., 2014). Mothers were asked the best response to the visual geometric design with a missing piece and the responses recorded. Raw scores were utilized for analysis.

### 3.5 Data entry and analysis

The Data Coordinating Centre (DCC) of the MAL‐ED study developed a double data entry database application. Individual forms were checked for completeness and accuracy of the data by site data supervisors and then data entered into the application. The discrepancies between first and second data entry were cleared by data supervisors after confirming from the forms. Missing values were minimal as data collection and checking were rigorous.

### 3.6 Statistical analysis

Continuous and categorical variables were summarized using mean (SD) and count (proportion), respectively. Variables summarized included SES (WAMI), maternal education, maternal cognition, maternal depression, parity, education of head of family, number of rooms in the house, number of people living in the family, type of floor, type of roof and position of the cooking place/stove. For analysis, the 16‐item, one factor SRQ was used for maternal depression as it was stronger psychometrically than the SRQ‐20 (Pendergast et al., 2014).

Multivariable linear regression models were used to identify the factors associated with HOME score for each time point. Linear mixed effect model was used to find the effect of WAMI and maternal factors on HOME score over a time period. Linear mixed effect model accounts for the correlation between the repeated measurements of same individual and different numbers of measurements per subject. The model allowed for a random intercept for each child. The effect of WAMI components on HOME subdomains was evaluated in a separate model. The effect of confounding can be adjusted by including the confounding variables into the model in addition to the covariates (Cook & Ranstam, 2017; Pourhoseingholi, Baghestani, & Vahedi, 2012). Thus, by including the gender and parity into the model, we adjusted for the confounding effects of gender and parity on HOME score. The estimated beta coefficients were reported with 95% confidence interval, and statistical significance was set at the 5% level. STATA 13. (StataCorp, College station, TX, USA) was used for data analysis.

## 4 RESULTS

**FIGURE 1:**
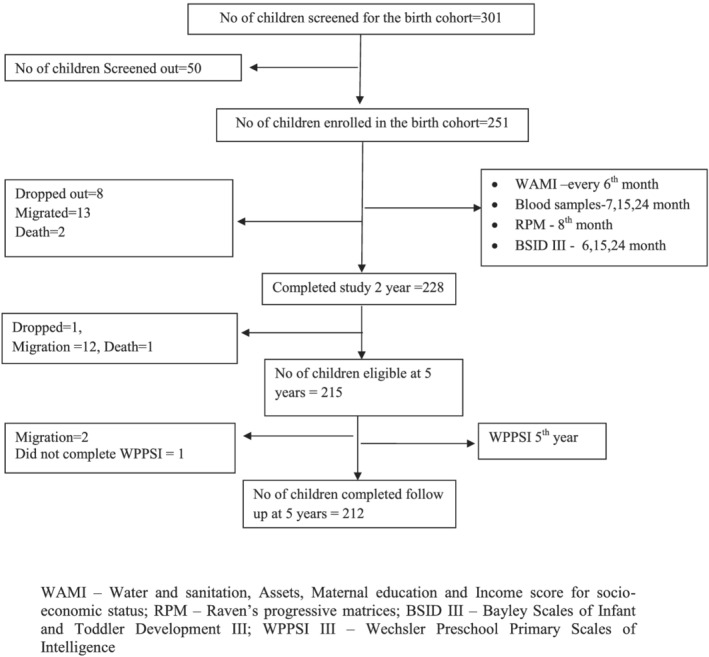
Flowchart depicting the birth cohort follow‐up

In the original cohort, 251 children were enrolled at birth after visiting 301 mothers. Fifty children were not enrolled due to the following reasons: pre‐existing plans for migration (*n* = 5), multiple pregnancy (*n* = 1), another child already enrolled in study (*n* = 8), medical co‐morbidities in children (*n* = 7), unavailability of mother (*n* = 9), combination of two or more of the above mentioned reasons (*n* = 10) and mothers refused participation (*n* = 10). Subsequently, 235 children participated at 6 months, 228 children at 2 years of age and 218 children at 3 years of age. The loss to follow‐up was mainly due to migration out of the study population (Figure 1).

More than 80% of the households recruited in the cohort had a monthly income of less than 5 000 INR (Table 1). Educational level of the head of the household was 6.81 years ranging from 0 to 18 years. More than 80% of families utilized public tap for water source, and more than 50% had no toilet facility. The mean family size in the birth cohort was 5.3, and participants were predominantly girls (56% at birth). The average birth weight (SD) for the cohort was 2.8 (0.44) kg (John et al., 2014).

**TABLE 1:** Basic characteristics of the birth cohort established in Vellore in 2010

Variables	6 months (*n* = 235)	24 months (*n* = 228)	36 months (*n* = 218)	*P* value^a^
Sex (%)	Female: 129 (54.9%)	123 (53.9%)	116 (53.3%)	
HOME score, mean (SD)	40.8 (3.24)	40.2 (3.42)	40.3 (3.18)	<0.001
Emotional and verbal Responsivity of caregiver, mean (SD)	10.6 (1.10)	11.0 (0.16)	11.0 (0.07)	<0.001
Avoidance of restriction and punishment, mean (SD)	4.7 (0.52)	4.2 (0.42)	4.1 (0.46)	<0.001
Caregiver promotes child development, mean (SD)	4.7 (0.69)	5.0 (0.15)	5.0 (0.20)	<0.001
Organization of physical and temporal environment, mean (SD)	10.3 (1.37)	9.6 (1.54)	9.5 (1.49)	<0.001
Provision of appropriate play materials, mean (SD)	2.2 (0.7)	2.4 (0.49)	2.7 (0.48)	<0.001
Opportunities for variety in daily stimulation, mean (SD)	5.4 (1.47)	5.9 (1.58)	5.8 (1.56)	<0.001
Cleanliness of child, mean (SD)	2.9 (0.43)	2.3 (0.95)	2.3 (0.85)	<0.001
WAMI standardized score, mean (SD)	0.5 (0.15)	0.5 (0.16)	0.5 (0.17)	<0.001
Improved sanitation score				<0.001^b^
1–4	124 (53.4%)	108 (47.6%)	51 (45.5%)	
5–8	108 (46.6%)	119 (52.4%)	61 (54.5%)	
Assets score				<0.001^b^
1–4	168 (72.4%)	125 (55.1%)	58 (51.8%)	
5–8	64 (27.6%)	102 (44.9%)	54 (48.2%)	
Maternal education: years, mean (SD)	6.9 (3.79)			
Income in INR				<0.001^c^
500–5000	191 (82.3%)	142 (62.6%)	54 (48.2%)^d^	
5 001–10 000	39 (16.8%)	71 (31.3%)	45 (40.2%)	
10 001–15 000	1 (0.4%)	9 (4.0%)	11 (9.8%)	
Highest through 15 000	1 (0.4%)	5 (2.2%)	2 (1.8%)	
Maternal depression (SRQ)score, mean (SD)	4.5 (3.73)	4.0 (3.31)	3.8 (3.53)	<0.001
Maternal cognition raw score, mean (SD)	43.9 (10.49)			
Head of household education: Years, mean (SD)	4.8 (3.86)			
Floor type, *N* (%)				0.085^c^
Cement floor	193 (83.5%)^e^	188 (83.2%)^f^	100 (89.3%)^d^	
Ceramic floor	23 (10%)	28 (12.4%)	12 (10.7%)	
Mud/dung floor	15 (6.5%)	10 (4.4%)	0 (0%)	

Abbreviations: HOME, Home Observation for the Measurement of the Environment; INR, Indian rupee; SD, Standard deviation; SRQ, Self‐Reporting Questionnaire; WAMI measure, Measure of access to improved **W**ater and sanitation, **A**ssets, **M**aternal education and total household **I**ncome.

^a^ *P* value calculated using repeated measures ANOVA.

^b^ Cochran Q test for correlated proportions.

^c^ Chi‐square test.

^d^ *N* = 112.

^e^ *N* = 231.

^f^ *N* = 226.

Characteristics of home environment and their changes are summarized in Table.1. Internal consistency of the 48‐item HOME was 0.610, 0.690 and 0.678 at 6, 24 and 36 months of age. Average HOME scores did not significantly vary over time. HOME subdomains varied over time, and the change between 6 and 36 months was significant for all subdomains. Emotional and verbal responsivity of caregiver, caregiver promotes child development component and provision of appropriate play material have improved over time; however, avoidance of restriction and punishment, organization of physical environment and cleanliness of the child have worsened over time. There was an improvement in the overall WAMI score for SES over time, the 3‐year WAMI score significantly better than the 6‐month score (paired *t* test; *t* = 7.2, *p* ≤ 0.001). Components of WAMI such as water and sanitation score (*t* = 2.2, *p* = 0.028), assets score (*t* = 10.01, *p* ≤ 0.001) and reported income score (*t* = 8.09, *p* ≤ 0.001) improved between 6‐ and 36‐month measurements except maternal education (*t* = 1.41, *p* = 0.16).

The WAMI score was the single biggest predictor for HOME environment across all age groups, with the relationship becoming stronger over time, beta (95% CI) being 1.728 (−1.186–4.642), 6.390 (3.651–9.129) and 9.843 (5.652–14.034), adjusted for other co‐variates, at 6, 24 and 36 months, respectively. Breaking down WAMI components, adjusted analyses revealed the number of assets a significant predictor for 6, 24 and 36 months HOME score (Table 2). Maternal education was a significant factor at 24 months. Maternal depression was negatively associated with home environment at both 6 and 24 months, while parity significantly affected home environment in an incremental fashion at 6 and 24 months. Other factors such as type of floor, type of roof, overcrowding and position of cooking stove did not have any effect on the total HOME score.

**TABLE 2:** Estimates from linear regression model for the effect of WAMI components and maternal factors on HOME score at 6, 24 and 36 months among a birth cohort in urban Vellore

	6th month			24th month			36th month		
	Univariate	Multivariable		Univariate	Multivariable		Univariate	Multivariable	
	Beta	Beta (95% CI)	*P*‐value	Beta	Beta (95% CI)	*P*‐value	Beta	Beta (95% CI)	*P*‐value
Female	−0.367	−0.473 (−1.247, 0.302)	0.231	−0.125	−0.287 (−1.040, 0.465)	0.453	−0.091	−0.020 (−1.221, 1.181)	0.974
WAMI‐components									
Water and sanitation (1–8)	0.062	−0.111 (−0.319, 0.097)	0.295	**0**.**334** ^*^	0.100 (−0.098, 0.298)	0.321	**0**.**416** ^*^	−0.023 (−0.351, 0.305)	0.890
Assets (1–8)	**0**.**483** ^*^	**0**.**365 (0**.**114**, **0**.**616)**	**0**.**005**	**0**.**667** ^*^	**0**.**384 (0**.**158**, **0**.**611)**	**0**.**001**	**1**.**006** ^*^	**0**.**751 (0**.**377**, **1**.**125)**	**<0**.**001**
Maternal education (1–8)	**0**.**396** ^*^	−0.064 (−0.340, 0.212)	0.648	**0**.**810** ^*^	**0**.**408 (0**.**153**, **0**.**663)**	**0**.**002**	**0**.**808** ^*^	0.376 (−0.034, 0.786)	0.072
Income (1–8)	0.172	−0.072 (−0.327, 0.183)	0.578	**0**.**487** ^*^	−0.135 (−0.380, 0.110)	0.278	**0**.**636** ^*^	0.137 (−0.214, 0.487)	0.440
Parity									
1	Ref	Ref		Ref	Ref		Ref	Ref	
2	**−1**.**266** ^*^	**−1**.**267 (−2**.**213**, **−0**.**320)**	**0**.**009**	**−1**.**166** ^*^	**−1**.**030 (−1**.**946**, **−0**.**114)**	**0**.**028**	−0.431	0.319 (−1.217, 1.856)	0.681
3	**−1**.**682** ^*^	**−1**.**291 (−2**.**402**, **−0**.**179)**	**0**.**023**	**−2**.**320** ^*^	**−1**.**563 (−2**.**624**, **−0**.**502)**	**0**.**004**	−0.986	0.139 (−1.602, 1.880)	0.875
>=4	**−3**.**015** ^*^	**−2**.**805 (−4**.**213**, **−1**.**397)**	**<0**.**001**	**−4**.**260** ^*^	**−2**.**986 (−4**.**383**, **−1**.**589)**	**<0**.**001**	**−2**.**875** ^*^	−0.217 (−2.454, 2.020)	0.848
Maternal cognition	**0**.**067** ^*^	0.039 (−0.004, 0.082)	0.078	**0**.**084** ^*^	0.017 (−0.024, 0.059)	0.417	**0**.**081** ^*^	0.005 (−0.063, 0.073)	0.879
Maternal depression	**−0**.**225** ^*^	**−0**.**193 (−0**.**298**, **−0**.**087)**	**<0**.**001**	**−0**.**247** ^*^	**−0**.**180 (−0**.**295**, **−0**.**065)**	**0**.**002**	**−0**.**189** ^*^	−0.084 (−0.264, 0.097)	0.359
Head of household education (Yrs)	0.077	‐		**0**.**243** ^*^	‐		**0**.**221** ^*^	‐	
Cement/ceramic tiles floor	1.140	0.536 (−1.097, 2.169)	0.519	1.199	0.097 (−1.731, 1.925)	0.917	‐	‐	

*Note*: 95% CI not provided for univariate beta. Significant associations in unadjusted and adjusted analyses are presented in bold letters.

Abbreviations: HOME, Home Observation for the Measurement of the Environment; WAMI measure, Measure of access to improved **W**ater and sanitation, **A**ssets, **M**aternal education and total household **I**ncome.

^*^ ***p* < 0**.**05**.

Modelling for change in the HOME score over time, the WAMI score for SES [Beta (95% CI): 6.597 (4.741–8.454)] was the single biggest predictor in the linear mixed effect model. Analyzing the components of WAMI, both the number of assets and maternal education predicted the change of HOME environment over time (Table 3).

**TABLE 3:** Estimates from the linear mixed effects model for the effect of WAMI components on HOME over time among a birth cohort in urban Vellore

Factors	Univariate		Multivariable	
	Beta (95% CI)	*P* value	Beta (95% CI)	*P* value
Female	−0.222 (−0.896, 0.453)	0.520	−0.287 (−0.823, 0.249)	0.294
WAMI components				
Water and sanitation	**0**.**169 (0**.**014**, **0**.**324)**	**0**.**032**	0.029 (−0.108, 0.166)	0.677
Assets	**0**.**542 (0**.**399**, **0**.**686)**	**<0**.**001**	**0**.**349 (0**.**193**, **0**.**504)**	**0**.**000**
Maternal education	**0**.**646 (0**.**480**, **0**.**813)**	**<0**.**001**	**0**.**262 (0**.**077**, **0**.**446)**	**0**.**005**
Income	0.160 (−0.002, 0.323)	0.053	−0.049 (−0.205, 0.107)	0.541
Parity				
1	Ref			
2	**−1**.**007 (−1**.**757**, **−0**.**257)**	**0**.**009**	**−0**.**935 (−1**.**593**, **−0**.**277)**	**0**.**005**
3	**−1**.**675 (−2**.**532**, **−0**.**818)**	**<0**.**001**	**−1**.**106 (−1**.**869**, **−0**.**344)**	**0**.**004**
>=4	**−3**.**332 (−4**.**354**, **−2**.**310)**	**<0**.**001**	**−2**.**444 (3**.**424**, **−1**.**464)**	**0**.**000**
Maternal cognitive	**0**.**078 (0**.**047**, **0**.**108)**	**<0**.**001**	0.024 (−0.006, 0.054)	0.115
Maternal depression	**−0**.**180 (−0**.**263**, **−0**.**098)**	**<0**.**001**	**−0**.**165 (−0**.**238**, **−0**.**092)**	**0**.**000**
Cement/ceramic tiles	−1.188 (−2.549, 0.173)	0.087	−0.568 (−1.676, 0.541)	0.316
Month	**−0**.**019 (−0**.**034**, **−0**.**004)**	**0**.**013**	**−0**.**048 (−0**.**068**, **−0**.**027)**	**0**.**000**

*Note*: Significant associations in unadjusted and adjusted analyses are presented in bold letters.

Abbreviations: HOME, Home Observation for the Measurement of the Environment; WAMI measure, Measure of access to improved **W**ater and sanitation, **A**ssets, **M**aternal education and total household **I**ncome.

Analyzing WAMI and HOME components together in a linear mixed effects model, maternal depression was negatively associated with parental emotional and verbal responsivity, punishment avoidance, provision of appropriate play materials, daily stimulation opportunities of the child and child's cleanliness over time (Table 4). Maternal cognition had a positive association with caregiver responsivity, child development promotion and physical organization of home over time. When corrected for other maternal factors, maternal education was positively associated with daily stimulation, and physical organization, but negatively associated with caregiver development promotion and emotional and verbal responsivity. Number of assets under the WAMI score was associated with punishment avoidance, physical organization and provision of appropriate play materials over time.

**TABLE 4:** Estimates from the linear mixed effects model for the effect of WAMI components on HOME subdomains over time among a birth cohort in urban Vellore

	Emotional and verbal Responsivity of caregiver			Avoidance of restriction and punishment			Caregiver promotes child development			Organization of Physical and Temporal Environment		
	Univariate Beta	Multivariate		Univariate Beta	Multivariate		Univariate Beta	Multivariate		Univariate Beta	Multivariate	
		Beta (95%CI)	*P*‐value		Beta (95%CI)	*P*‐value		Beta (95%CI)	*P*‐value		Beta (95%CI)	*P*‐value
Female	−0.040	−0.064 (−0.179, 0.052)	0.281	−0.027	−0.040 (−0.122, 0.042)	0.339	−0.055	−0.068 (−0.142, 0.006)	0.071	0.005	−0.041 (−0.293, 0.211)	0.751
**WAMI components**												
Water and sanitation	−0.021	−0.025 (−0.056, 0.005)	0.106	−0.004	−0.016 (−0.037, 0.006)	0.155	−0.005	−0.004 (−0.024, 0.015)	0.674	0.127^*^	0.054 (−0.009, 0.117)	0.092
Assets	0.014	0.021 (−0.015 ,0.057)	0.249	**0**.**031** ^*^	**0**.**026 (0**.**001**, **0**.**051)**	**0**.**041**	0.013	0.017 (−0.006 ,0.040)	0.142	**0**.**270** ^*^	**0**.**176 (0**.**104**, **0**.**247)**	**<0**.**001**
Maternal education	−0.008	**−0**.**046 (−0**.**086**, **−0**.**006)**	**0**.**023**	0.011	−0.010 (−0.039, 0.018)	0.481	0.000	**−0**.**027 (−0**.**053**, **−0**.**002)**	**0**.**035**	**0**.**264** ^*^	**0**.**118 (0**.**031**, **0**.**204)**	**0**.**008**
Income	−0.008	−0.020 (−0.057, 0.017)	0.282	0.028^*^	0.017 (−0.008, 0.043)	0.177	0.000	−0.006 (−0.030, 0.017)	0.607	0.122^*^	0.023 (−0.047, 0.094)	0.513
Parity												
2	**−0**.**127** ^*^	**−0**.**167 (−0**.**309**, **−0**.**025)**	**0**.**021**	−0.078	−0.069 (−0.17, 0.032)	0.180	**−0**.**097** ^*^	**−0**.**118 (−0**.**208**, **−0**.**027)**	**0**.**011**	−0.093	−0.051 (−0.360, 0.257)	0.744
3	−0.017	−0.048 (−0.213, 0.116)	0.565	−0.115^*^	−0.110 (−0.227, 0.007)	0.064	−0.011	−0.024 (−0.129, 0.081)	0.658	**−0**.**821** ^*^	**−0**.**542 (−0**.**900**, **−0**.**184)**	**0**.**003**
>=4	−0.063	−0.185 (−0.396, 0.026)	0.086	0.004	0.015 (−0.136, 0.165)	0.849	−0.023	−0.087 (−0.222, 0.048)	0.205	**−1**.**398** ^*^	**−0**.**858 (−1**.**319**, **−0**.**398)**	**<0**.**001**
Maternal cognition	**0**.**005** ^*^	**0**.**008 (0**.**002**, **0**.**015)**	**0**.**014**	0.002	0.001 (−0.004, 0.006)	0.686	**0**.**004** ^*^	**0**.**006 (0**.**002**, **0**.**010)**	**0**.**004**	0.023^*^	−0.002 (−0.016, 0.012)	0.826
Maternal depression	**−0**.**031** ^*^	**−0**.**03 (−0**.**050**, **−0**.**017)**	**<0**.**001**	**−0**.**026** ^*^	**−0**.**021 (−0**.**033**, **−0**.**010)**	**<0**.**001**	−0.008	−0.008 (−0.018, 0.003)	0.153	−0.022	−0.018 (−0.052, 0.015)	0.283
Cement/ceramic tiles floor	−0.040	−0.030 (−0.268, 0.209)	0.807	−0.037	−0.013 (−0.183, 0.156)	0.878	0.021	0.024 (−0.128, 0.177)	0.753	−0.660	−0.317 (−0.838 ,0.204)	0.233
Month	**0**.**014** ^*^	**0**.**016 (0**.**010**, **0**.**021)**	**<0**.**001**	**−0**.**020** ^*^	**−0**.**024 (−0**.**028**, **−0**.**021)**	**<0**.**001**	**0**.**010** ^*^	**0**.**012 (0**.**009**, **0**.**015)**	**<0**.**001**	**−0**.**029** ^*^	**−0**.**047 (−0**.**056**, **−0**.**038)**	**<0**.**001**

	Provision of appropriate play materials			Opportunities for variety in daily stimulation			Cleanliness of child		
	Univariate Beta	Multivariate		Univariate Beta	Multivariate		Univariate Beta	Multivariate	
		Beta (95%CI)	*P*‐value		Beta (95%CI)	*P*‐value		Beta (95%CI)	*P*‐value
Female	−0.076	**−0**.**095 (−0**.**190**, **−0**.**001)**	**0**.**048**	−0.124	−0.086 (−0.355, 0.183)	0.531	0.109	0.101 (−0.032, 0.235)	0.138
**WAMI components**									
Water and sanitation	0.010	−0.008 (−0.033, 0.017)	0.520	0.047	0.007 (−0.061, 0.075)	0.844	0.042^*^	0.020 (−0.015, 0.054)	0.264
Assets	**0**.**052** ^*^	**0**.**044 (0**.**015**, **0**.**073)**	**0**.**003**	**0**.**146** ^*^	0.075 (−0.003, 0.152)	0.058	0.055^*^	0.012 (−0.027, 0.052)	0.541
Maternal education	**0**.**040** ^*^	0.001 (−0.031, 0.034)	0.933	**0**.**258** ^*^	**0**.**187 (0**.**094**, **0**.**279)**	**<0**.**001**	0.083^*^	0.034 (−0.012, 0.080)	0.146
Income	**0**.**029** ^*^	0.001 (−0.028, 0.031)	0.927	0.028	−0.049 (−0.126, 0.028)	0.211	0.028	−0.011 (−0.052, 0.029)	0.574
Parity									
2	−0.030	0.000 (−0.116, 0.117)	0.996	−0.509^*^	−0.503 (−0.834, −0.173)	0.003	−0.043	−0.019 (−0.183, 0.145)	0.818
3	−0.112	−0.071 (−0.205, 0.064)	0.304	−0.450^*^	−0.246 (−0.629, 0.137)	0.209	−0.154	−0.065 (−0.255, 0.125)	0.503
>=4	**−0**.**302**	**−0**.**329 (−0**.**502**, **−0**.**156)**	**<0**.**001**	**−0**.**980** ^*^	**−0**.**561 (−1**.**054**, **−0**.**069)**	**0**.**026**	**−0**.**594** ^*^	**−0**.**438 (−0**.**682**, **−0**.**194)**	**<0**.**001**
Maternal cognition	0.003	0.000 (−0.005, 0.005)	0.996	0.029^*^	0.005 (−0.010, 0.020)	0.516	**0**.**010** ^*^	0.005 (−0.002, 0.012)	0.193
Maternal depression	**−0**.**019** ^*^	**−0**.**014 (−0**.**028**, **−0**.**001)**	**0**.**036**	**−0**.**050** ^*^	**−0**.**042 (−0**.**079**, **−0**.**006)**	**0**.**022**	**−0**.**027** ^*^	**−0**.**022 (−0**.**040**, **−0**.**003)**	**0**.**021**
Cement/ceramic tiles floor	0.033	0.127 (−0.068, 0.323)	0.201	−0.210	−0.114 (−0.671, 0.443)	0.689	−0.296^*^	−0.221 (−0.497, 0.055)	0.117
Month	**0**.**013** ^*^	**0**.**004 (0**.**001**, **0**.**008)**	**0**.**077**	**0**.**013** ^*^	**0**.**015 (0**.**005**, **0**.**025)**	**0**.**003**	**−0**.**021** ^*^	**−0**.**025 (−0**.**030**, **−0**.**020)**	**<0**.**001**

*Note*: 95% CI not provided for univariate beta. Significant associations in unadjusted and adjusted analyses are presented in bold letters.

Abbreviation: WAMI measure, measure of access to improved **W**ater and sanitation, **A**ssets, **M**aternal education and total household **I**ncome.

* *P* < 0.05.

## 5 DISCUSSION

This prospective community‐based birth cohort follow‐up study from Vellore provides short term trends and predictors of home environment in early childhood in a semi‐urban slum in South India. To our best knowledge, this is the first such report from India. There was only a minimal drop out of recruited children for this longitudinal study. The single strongest predictor for home environment at each time point in early childhood and for its change over time was the SES.

In this birth cohort, the SES significantly improved in lieu of improved sanitation, assets and reported income over 3 years. Economic surveys in India also have shown similar improvement in SES over time especially in southern India where the current birth cohort study was conducted (Ohlan, 2013). This trend is also in concurrence with the World Bank report of improving general economic status across the globe (Renton, Wall, & Lintott, 2012).

Overall HOME environment score did not change, but individual subdomains significantly changed over time. The HOME subdomains of physical organization, avoidance of punishment and cleanliness of the child have worsened over time. Factors such as mobility of the child, the presence of other caregivers in the family, opportunities for exploration and semi‐urban slum nature of the environment might have influenced the above HOME factors. Other HOME subdomains of emotional responsivity of caregiver, caregiver promoting development, play material provision and opportunities for variety have improved between 6 and 36 months of age probably due to increased exploration and play of older children. In addition to SES, family factors such as the number of resident people, the number of under‐fives in the family and companionship dynamics including father's contribution to the family care can affect the home environment (Bradley & Corwyn, 2005; Sarsour et al., 2011). It is possible that some of these factors would have also changed in this birth cohort.

The SES score was the single largest predictor for the home environment at 6, 24 and 36 months of age. It has been shown that poverty affects the home environment, cutting across ethnic barriers in both high income and low‐ and middle‐income countries (Bradley, Corwyn, McAdoo, & García Coll, 2001; Yunus & Dahlan, 2013). SES can affect the home environment not only in terms of physical factors such as a safe and stimulating milieu, availability of toys and other play materials and exposure to a variety of enriching experiences but also in parental responsiveness and discipline practices (Andrade et al., 2005; Bradley & Corwyn, 2005). Suboptimal home environments can predispose exposure to industrial chemicals such as lead, arsenic and methylmercury which can lead to neurodevelopmental disorders such as cerebral palsy, autism as well as subclinical brain dysfunction causing learning and behavioural problems (Grandjean & Landrigan, 2006). Home physical environmental factors of early childhood such as safety, cleanliness and crowding have shown concurrent and predictive effects on child development, cognition, academic performance and executive functions (Delgado, Ramirez‐Cardich, Gilman, et al., 2002; Solari & Mare, 2012). Home factors can mediate the effect of SES on motor development, vocabulary acquisition and executive function in children and can also outweigh the effect of SES (Ferreira et al., 2018; Lohndorf et al., 2018; Sarsour et al., 2011). In the current study, analyzing SES further, the number of assets, was a consistent predictor for home environment at 6, 24 and 36 months and for the change in the home environment, reflecting availability of appropriate play materials also concurrently for children. Other physical factors such as improved water supply and reported income neither affected the environment nor its change.

As home environment has both physical and interactive components, parental well‐being and parenting styles also can affect the child's environment. In the current study, maternal factors were significant predictors for not only home environments at 6, 24 and 36 months but also for the environment change over time. Maternal depression was associated with 6‐ and 24‐month home environments, whereas maternal education level was associated with 24‐month environment. Similarly parity of the mother negatively affected home environments at 6 and 24 months of age in an incremental fashion, probably indicating mother being pre‐occupied with other siblings and needs of the family. Analyzing both WAMI and HOME components further, maternal factors (maternal cognition, education and depression) were associated with relational HOME components (emotional and verbal caregiver responsivity, caregiver promotes child development and opportunities for variety in daily stimulation) more, while physical HOME components (organization of physical and temporal environment and provision of appropriate play materials) were associated with the number of assets. The mother is the primary caregiver of the child in this birth cohort, and her cognitive and mental health status affect relational and interactive home factors. Other studies have also reported the importance of maternal factors in parenting styles and for creating an organized, responsive and stimulating child care environment (Andrade et al., 2005; Bradley et al., 1994; Pachter et al., 2006). Maternal education can mediate the effect of home stimulation on child cognition (Andrade et al., 2005) and relational factors the effect of physical home components on child cognition (Evans et al., 2010). When multiple stress factors are present such as poverty and preterm birth, protective factors such as sensitive and responsive parenting can help in the development of early resiliency in children (Bradley et al., 1994). Maternal depression can directly impact child behaviour, an effect more pronounced with low maternal education, fewer assets and indigenous ethnicity (Flynn et al., 2017). The importance of maternal wellbeing in early childhood stimulation has been well‐recognized by the WHO (Daelmans et al., 2017). This study concurs with that existing evidence in showing maternal factors impacting early childhood home environment consistently and new evidence of maternal features also affecting a change in this early childhood home environment.

The Rashtriya Bal Swasthya Karyakram (RBSK) launched under National Rural Health Mission by the Indian government aims to identify and correct birth defects, nutritional deficiencies, treatable diseases and developmental delays including disability (https://rbsk.gov.in) (Singh, Kumar, Mishra, Khera, & Srivastava, 2015). The overall programme is inclusive to need‐based interventions but must move towards active child development promotion as suggested by the WHO, with additional maternal well‐being component (World Health Organization, 2018).

There are limitations in the current study. There have been dropouts in the follow‐up phase of the study. An optimal examination of the home environment is challenging, but due care was taken in this study to use culturally appropriate HOME scale after piloting (Jones et al., 2017). The WAMI scale used to measure SES has been developed during the course of the larger multi‐national study and uses a normalized score (Psaki et al., 2014). Despite these limitations, the strengths include that this is a community‐based study with follow‐up of a birth cohort. Measures and methods used in this study have been extensively reviewed and approved by international experts, as this study was part of a large multi‐national study.

## 6 CONCLUSIONS

The current study confirmed the persistent association of SES and maternal factors on early childhood home environments, as well as showed new evidence of these factors being associated with home environment changes. Maternal factors predicted relational home environmental change over time. Maternal well‐being needs to be enquired and supported at well‐baby clinics and maternal education supported for holistic socio‐economic development planning. Parent–child interactions can improve by implementing the ‘Care for child development package’ developed by the WHO (World Health Organization, 2012).

Future studies can explore ways to improve mother–child interactions in different cultural and socio‐economic backgrounds. Cultural and parenting practices of the region also need to be explored prudently before incorporating scientifically valid interventions to improve early childhood experiences.

## FINANCIAL SUPPORT

The Etiology, Risk Factors and Interactions of Enteric Infections and Malnutrition and the Consequence for Child Health and Development Project (MAL‐ED) is carried out as a collaborative project supported by the Bill and Melinda Gates Foundation, the Foundation for the NIH and the National Institutes of Health/Fogarty International Center.

## CONFLICT OF INTERESTS

None.

## AUTHOR CONTRIBUTION

Drs. GK, SJ, AB, VRM, RR and BK are involved in the birth cohort study planning, recruitment and follow‐up as well as in the planning, analysis, write‐up and correction of the current study. Dr. AK analyzed data and are involved in the study write‐up and corrections. Mr. KR was the data co‐ordinator who initially analyzed the data. All authors approved the final manuscript and consent for publication.

## ETHICS APPROVAL

This study was conducted according to the guidelines laid down in the Declaration of Helsinki and all procedures involving human subjects/patients were approved by the Institutional Review Board, Christian Medical College, Vellore. Written informed consent was obtained from all participants' parents.

## AVAILABILITY OF DATA AND MATERIALS

The datasets used and analyzed during the current study are available from the corresponding author on reasonable request.
